# Dying of Stress: Chemotherapy, Radiotherapy, and Small-Molecule Inhibitors in Immunogenic Cell Death and Immunogenic Modulation

**DOI:** 10.3390/cells11233826

**Published:** 2022-11-29

**Authors:** Kellsye P. Fabian, Joshua T. Kowalczyk, Sandy T. Reynolds, James W. Hodge

**Affiliations:** Center for Immuno-Oncology, Center for Cancer Research, National Cancer Institute, National Institutes of Health, Bethesda, MD 20892, USA

**Keywords:** immunogenic cell stress, immunogenic cell death, immunogenic modulation, chemotherapy, radiotherapy, small molecule inhibitor, immunotherapy, combination therapy

## Abstract

Innovative strategies to re-establish the immune-mediated destruction of malignant cells is paramount to the success of anti-cancer therapy. Accumulating evidence suggests that radiotherapy and select chemotherapeutic drugs and small molecule inhibitors induce immunogenic cell stress on tumors that results in improved immune recognition and targeting of the malignant cells. Through immunogenic cell death, which entails the release of antigens and danger signals, and immunogenic modulation, wherein the phenotype of stressed cells is altered to become more susceptible to immune attack, radiotherapies, chemotherapies, and small-molecule inhibitors exert immune-mediated anti-tumor responses. In this review, we discuss the mechanisms of immunogenic cell death and immunogenic modulation and their relevance in the anti-tumor activity of radiotherapies, chemotherapies, and small-molecule inhibitors. Our aim is to feature the immunological aspects of conventional and targeted cancer therapies and highlight how these therapies may be compatible with emerging immunotherapy approaches.

## 1. Introduction

Conventional anti-cancer therapies were originally developed to negatively impact rapidly dividing malignant cells while limiting damage to normal tissues. Typically, these therapeutic regimens induce DNA damage or disrupt DNA repair and cell division machineries, resulting in tumor cell death and debulking of cancer lesions [1]. In addition to these cytotoxic and cytostatic effects, some standard-of-care therapies such as radiotherapy, select chemotherapies, and small-molecule inhibitors (SMIs) also trigger cell stress that results in responses spanning from immunogenic cell death to immunogenic modulation. Immunogenic cell death (ICD), wherein dying cells release signals that prompt dendritic cells (DCs) to present tumor antigens to T cells, culminates in the activation and development of immunological memory [2,3]. If the therapy-induced cell stress does not result in cell death, the surviving tumor cells may be sensitized to immune cell targeting via immunogenic modulation, which includes upregulated antigen presentation, pro-apoptotic signaling molecules, and immune-cell-engaging molecules [4]. As immunotherapy emerges as a new pillar among anti-cancer therapies, it is important to recognize the immunogenic properties of conventional anti-cancer therapies and identify how these different treatment modalities can be synthesized together in the clinic. In this review, we discuss preclinical data on ICD and immunogenic modulation mediated by chemotherapy, radiotherapy, and SMIs and clinical data on the immunostimulatory effects of these conventional cancer treatments in the context of combination regimens with immunotherapy.

### 1.1. Immunogenic Cell Death

**Figure 1:**
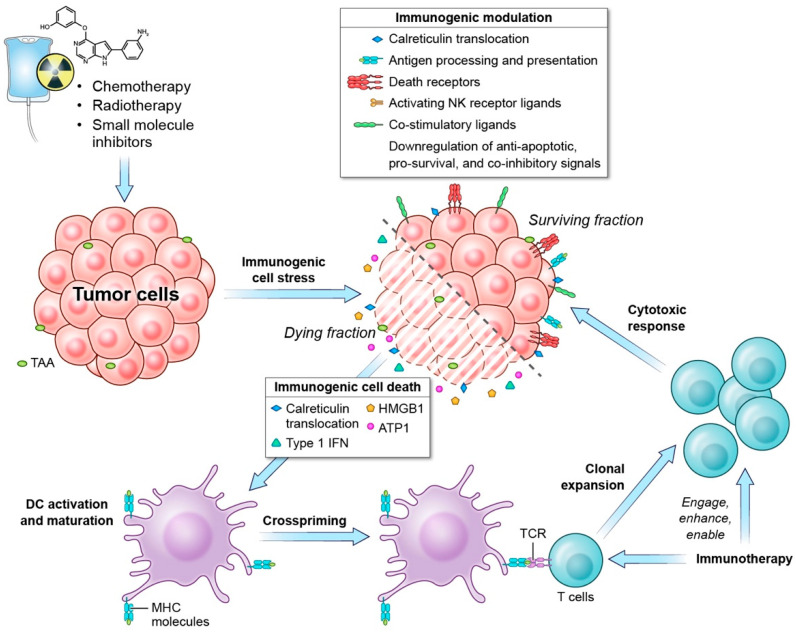
Radiotherapy, chemotherapy, and small-molecule inhibitors induce immunogenic cell stress resulting in immunological responses spanning from immune modulation to immunogenic cell death. Radiotherapy, select chemotherapy, and small-molecule inhibitors can induce immunogenic cell death, which is characterized by the release of DAMPs that promote antigen presenting cells to phagocytose the dying cells, process tumor antigens, and present tumor antigens to T cells. Tumor cells that were not eradicated by radiotherapy, chemotherapy, or small-molecule inhibitors undergo immunogenic modulation wherein the tumor phenotype is altered such that the malignant cells become more sensitized to T cell targeting. Combining radiotherapy, chemotherapy, and small-molecule inhibitors with immunotherapies that can engage, enhance, and enable effector T cells may result in improved immune-mediated eradication of neoplastic cells. DAMP: damage-associated molecular patterns; TAA: tumor-associated antigen; HMGB1: high-mobility group box 1; ATP: adenosine triphosphate; IFN: interferon; MHC: major histocompatibility complex; TCR: T-cell receptor.

ICD is defined as “a form of regulated cell death that is sufficient to activate an adaptive immune response in immunocompetent hosts” [5]. ICD is a spatiotemporally defined process that involves the induction of the cell-stress response pathways and entails the release of antigens and danger signals from the dying cell that initiates the immune response (Figure 1). The activation of endoplasmic reticulum (ER) and/or reactive oxygen species (ROS) stress responses is essential for the initiation of ICD [6], and ICD inducers can be classified according to how they elicit ER stress. Most ICD inducers are Type I ICD inducers that instigate cell death by targeting organelles or molecules that are not directly associated with the ER but cause “collateral” ER stress effects [7,8]. Type II ICD inducers, on the other hand, directly target the ER to trigger ER stress and induce cell death [7,8]. Depending on dose and schedule, several anti-cancer therapies have been shown to cause ICD as a consequence of cell stress, and in this review, we concentrate on the immunogenic cell stress mediated by chemotherapy, radiotherapy, and SMIs. However, it should also be noted that other treatment regimens, such as oncolytic viruses [9], oncolytic peptides [10,11], the epithelial growth factor receptor (EGFR)-targeting monoclonal antibody cetuximab [12], and nanopulse stimulation [13], have also been shown to induce immunogenic cell stress.

The anti-cancer adaptive immune response associated with ICD relies on two major factors: (1) the antigenicity of the tumor cells and (2) the release of adjuvant signals from the dying tumor cells [2]. Generally, tumor antigens can be characterized as tumor neoantigens, which are products of non-synonymous mutations that are not covered by central tolerance, or tumor-associated antigens (TAAs), which are non-mutated antigens to which central tolerance is leaky or incomplete [14,15,16]. Epigenetic dysregulation of the cancer genome can additionally trigger the expression of human endogenous retroviruses (HERVs), resulting in tumor-specific or tumor-enriched HERV-derived antigens [17]. Some ICD inducers can cause mutations or introduce post-translational modifications that produce neoantigens or enhance the expression of neoantigens and TAAs [3,18,19,20]. 

The release of tumor antigens must be accompanied by pro-inflammatory signals in order to elicit an immune response that culminates in the effective immune-mediated killing of cancer cells [21]. Adjuvant signals generated during ICD come in the form of damage-associated molecular patterns (DAMPs) that play key roles in the recruitment and maturation of antigen presenting cells (APCs) [3,22]. Different ICD inducers generate different DAMPs due to variations in the cell-stress response activated by each ICD inducer [3,23]. Examples of DAMPs commonly generated during ICD include calreticulin (CRT), high mobility group box 1 (HMGB1), adenosine triphosphate (ATP), and type 1 interferons (IFN) [3,22,23]. CRT is an ER chaperone protein that translocates to the plasma membrane during ICD and provides an “eat me” signal to APCs [24,25,26,27]. The ligation of CRT to CD91 on APCs promotes phagocytosis of cellular debris and antigen presentation [24]. HMGB1, which is passively released by dying cells during ICD, binds to Toll-like receptor 4 (TLR4) on DCs, resulting in the efficient cross-presentation of antigens from the dying tumor cells [28]. ATP, which is actively released via exocytosis during ICD, provides a “find me” signal to DC precursors and macrophages [29]. Extracellular ATP interacts with purinergic receptor P2Y2 (P2RY2) and directs APC chemotaxis to the site of active ICD [30]. In addition, extracellular ATP can influence DC activation and antigen presentation via IL-1b, which is expressed following activation of the NLRP3 (NOD-, LRR-, and pyrin domain-containing protein (3) inflammasome downstream of ATP signaling through P2RX7 on DCs [31]. Type 1 IFN secretion can be driven by nucleic acid species from dying cells binding to different pattern recognition receptors (PRRs) [32]. Type I IFNs exert immunostimulatory effects, including the enhancement of the cytotoxic functions of cytotoxic T lymphocytes (CTLs) and natural killer (NK) cells [33,34], the promotion of cross-priming capacity of APCs [35], and the production of the T cell chemoattractant, CXCL10, by cancer cells [36]. Other DAMPs, such as annexin A1, heat shock protein (HSP) 70, and HSP90, have also been shown to be secreted or translocated by cells undergoing ICD [22,23]. Emission of these DAMPs according to a specific spatiotemporal order is essential for the recruitment and activation of innate and adaptive immune cells in the tumor lesion to mount an effective anti-cancer immune response [37].

### 1.2. Immunogenic Modulation

Cell stress induced by certain anti-cancer treatments does not always translate to cell death, but it can sensitize the surviving malignant cells to immune attack. Through the process of immunogenic modulation, therapies such as chemotherapy, radiotherapy, and SMIs alter the tumor phenotype to become more sensitive to CTL and NK cell killing [22,38]. Immunogenic modulation includes, but is not limited to, enhanced antigen presentation, increased pro-apoptotic signals, and changes in surface marker expression of the tumor cells (Figure 1) [4,22,38]. ICD and immunogenic modulation exist on a continuum and represent a spectrum of outcomes resulting from immunogenic cell stress [22].

Similar to ICD, immunogenic modulation requires antigenic determinants on malignant cells to trigger anti-tumor responses. In addition to promoting the expression of neoantigens and TAAs, immunogenic modulators upregulate different components of the antigen processing machinery and promote the expression of MHC class I [18,19,20,39,40]. This increase in antigen processing and presentation has been shown to be associated with enhanced sensitivity of the treated neoplastic cells to TAA-specific CTLs [20]. Since CTL-mediated killing may also be a form of ICD, the resulting anti-tumor effect following ICD or immunogenic modulation can trigger antigen cascade and generate new CTLs against other tumor antigens [41,42,43].

Immunogenic modulators also alter the tumor phenotype by promoting the expression of surface markers that make the tumor cells more susceptible to attack by innate and adaptive immune cells. For example, cell surface translocation of CRT, which acts as an “eat me” signal to APCs during ICD [24,25,26,27], also enables the myeloid cells to deliver stimulatory signals to NK cells [44]. Additionally, immunogenic modulators induce the expression of NK activating ligands on tumor cells such as MICA/B, ULPBs, and B7-H6 [45,46,47]. Furthermore, select immunogenic modulators also upregulate mannose-6-phosphate receptors (M6PR) on the tumor cell surface, which augment cell membrane permeability to granzyme B produced by activated NK cells and CTLs [48,49]. Tumor cells are also sensitized to immune-mediated killing via the upregulation of death receptors, such as Fas and TRAIL receptors, that can interact with death ligands expressed or secreted by CTLs and NK cells [50,51]. Immunogenic modulators also support CTLs through the induction of costimulatory molecules, including CD80, OX40L, and 41BBL [52,53]. Overall, these observations demonstrate that chemotherapy, radiotherapy, and SMIs have capabilities that allow for enhanced anti-tumor immunity that is distinct from but related to ICD [22]. 

### 1.3. Role of Immunogenic Cell-Stress Response in Anti-Tumor Response

Mechanistically, it is challenging to exactly elucidate to what extent ICD and immunogenic modulation contribute to improved disease outcomes in cancer patients. However, a body of evidence demonstrates that activation of downstream immune-related components of ICD and immunogenic modulation are correlated to therapeutic benefit in several cancer types. For example, the occurrence of the abscopal effect (see Section 3.1) with radiotherapy is good evidence of radiation-induced anti-tumor immunity that is linked to ICD [54]. Furthermore, high CRT exposure on malignant cells has been linked to enhanced immune activation and improved disease outcomes in patients with acute myeloid leukemia and ovarian cancer [44,55,56]. Esophageal squamous cell carcinoma patients who received pre-operative chemoradiotherapy were found to have elevated HMGB1 compared to patients who did not receive the immunomodulating treatment modality. Furthermore, in this patient cohort, elevated serum HMGB1 was associated with antigen-specific T cell responses and the degree of HMGB1 expression in the tumor microenvironment (TME) was correlated with improved patient survival [57]. On the other hand, loss-of-function mutations in the TLR4 allele have been associated with reduced progression-free survival and overall survival in patients with colorectal cancer receiving oxaliplatin treatment [58], shorter disease-free survival in patients with head and neck cancer receiving adjuvant systemic therapy including cisplatin and 5-fluoruracil [59], and decreased time-to-metastasis in patients with non-metastatic breast cancer undergoing surgery followed by anthracycline-based chemotherapy and local irradiation [28]. These data suggest that ICD and immunogenic modulation play a role in the anti-tumor response and can be exploited via combinatorial approaches, especially with immunotherapy, to improve disease outcomes in cancer patients. 

## 2. Chemotherapy and Immunogenic Cell Stress

### 2.1. Mechanisms of Chemotherapy-Induced Immunogenic Cell Stress

The use of chemotherapy as a treatment modality for cancer started in the mid-20th century and has remained one of the pillars of cancer therapy [60,61]. Currently, chemotherapy is a component of the first-line treatment option for several cancer types, including breast [62], small-cell lung and non-small-cell lung [63,64], colorectal [65], pancreatic [66], and bladder [67] cancer. As immunotherapy emerges as a new pillar of cancer therapy, identifying how immunotherapy might synthesize with chemotherapy in this new era is critical. Many chemotherapeutic agents have immunosuppressive side effects, such as depletion of effector cells [68]. Theoretically, this characteristic would make chemotherapeutic drugs incompatible with immunotherapy. Paradoxically, depending on dose and schedule, chemotherapy-induced lymphopenia may actually be beneficial for the expansion of tumor antigen-specific effector cells, especially in the context of vaccine treatment and adoptive cell transfer [4,69]. Chemotherapeutic agents also exert anti-cancer immunity by directly interacting with immune cell subsets causing the selective depletion of immunosuppressive cells and the activation of immune effector cells [70]. Lastly, chemotherapy induces immunogenic modulation and ICD, allowing for improved immune targeting of tumor cells [22].

Immunogenic modulation and ICD are consequences of cell stress; however, chemotherapeutic agents do not directly target the ER. Different conventional chemotherapeutic agents have diverse chemical structures and functions, yet their basic mechanisms involve targeting the rudimentary functions of cell division [71]. Chemotherapeutic agents classified as alkylating agents, antimetabolites, anti-tumor antibiotics, and topoisomerase inhibitors primarily target the DNA or DNA replication machinery, whereas mitotic inhibitors disrupt the microtubules [22,68]. These on-target effects also frequently result in inhibition of RNA transcription, protein translation, and cell replication, inducing ER stress response via secondary effects [22,72] that activate the protein kinase RNA (PKR)-like ER kinase (PERK) signaling pathway, which ultimately promotes the cell-surface translocation of CRT, an important DAMP involved in ICD and immunogenic modulation [73,74,75,76]. Notably, abrogating the ER stress response minimizes the immunogenicity of chemotherapy. Mutation or knockdown of PERK and its downstream molecular components such as eIF2a, caspase-8, BAP31, Bax, Bak, or SNAREs inhibits CRT translocation by anthracycline and oxaliplatin. Interestingly, anthracyclines continue to stimulate cell death despite depletion of PERK, caspase-8, or SNAREs but fail to induce ICD [76]. Furthermore, knockdown or blockade of CRT reduces DC-mediated phagocytosis and immune responses to tumor cells treated with anthracyclines [25]. In these studies, immunogenicity was restored via adsorption of recombinant CRT on the tumor cell surface [25,76]. 

The level of immunogenicity, which can range from immunogenic modulation to ICD, of a particular chemotherapy is associated with the intensity and kinetics of the ER stress response it induces [22,72,77]. Traditional chemotherapeutic agents that are considered bona fide ICD inducers include several anthracyclines such as doxorubicin, epirubicin, idarubicin, and mitoxantrone, as well as some alkylating agents such as cyclophosphamide and oxaliplatin [8]. Other chemotherapeutic agents do not induce ICD but are still immunogenic, albeit to lesser degrees. Non-ICD-inducing chemotherapies including, but not limited to, anti-metabolites (i.e., 5-fluorouracil, 5′-aza-2′deoxycytidine, and gemcitabine), mitotic inhibitors (docetaxel, vinorelbine, and paclitaxel), and alkylating agents (cisplatin, melphalan, and carboplatin) have been reported to promote immunogenic modulation and sensitize tumor cells to immune attack through phenotypic alterations, including increased tumor antigen expression and presentation, increased apoptotic signaling, upregulated expression of activating NK ligands, and augmented cell membrane permeability [4,22,38,78]. For instance, in studies exploring the effects of docetaxel and cisplatin/vinorelbine in diverse human cancer cell lines, it was shown that the chemotherapies enhanced TAA expression and upregulated components of the antigen processing and presentation machinery, including MHC-I, peptide transporters, chaperones, and immunoproteasome subunits [20,79]. Notably, blocking MHC-I was shown to decrease CTL-mediated killing of chemotherapy-treated tumor cells, establishing the role of upregulated MHC-I in the chemotherapy-induced immunogenic response. In addition, docetaxel and cisplatin/vinorelbine promoted pro-apoptotic signals and enhanced sensitivity to CTL-mediated killing, specifically via upregulation of Fas and ICAM-1 [20,79].

The characteristics (structural, chemical, mechanistic, etc.) that enable chemotherapy to induce ICD, immunogenic modulation, or both are currently unknown. Cisplatin, despite its similarity to the ICD-inducer oxaliplatin, is unable to induce the strong ER response needed for the surface exposure of CRT and thus is incapable of promoting bona fide ICD [80,81]. Nevertheless, cisplatin is capable of inducing immunogenic modulation such as the upregulation of MHC class I, M6PR, and Fas expression on tumor cells, thereby sensitizing the tumor cells to immune attack [48,82,83,84]. Meanwhile, some chemotherapies that are capable of promoting CRT translocation are incapable of inducing bona fide ICD [78]. For example, docetaxel treatment increased CTL-mediated killing of tumor cells via CRT translocation and increased antigen processing but did not induce DAMP (ATP or HMGB1) secretion required for ICD [20]. Even with the gaps in knowledge on what exactly dictates ICD versus immunogenic modulation, it appears that both are part of the same spectrum that results from immunogenic cell stress [22].

### 2.2. Chemotherapy-Induced Immunogenic Cell-Stress Response in Preclinical Models

The immunogenic properties of chemotherapy could be harnessed to potentiate the anti-tumor activity of immunotherapeutic regimens. Immune checkpoint inhibitors (ICIs) targeting CTLA-4 and the PD-1/PD-L1 axis have shown promising clinical activity in multiple cancer types, but so far only a limited population of patients benefits from these therapies [85]. Several preclinical findings suggest that chemotherapy and ICIs are complementary to one another and thus may be rational combination partners to elicit synergistic or additive anti-tumor effects. First, tumors with an inflamed phenotype have higher response rates to ICIs [86,87]. In several murine tumor models, chemotherapy, via ICD and/or direct effects on immune cells, has been shown to promote immune infiltration into the TME, thereby increasing the efficacy of ICIs [78,88,89,90,91]. One study indicated that immunogenic chemotherapy may be capable of converting immunologically cold lesions into hot tumors [88]. Pfirschke, et al. reported that autochthonous tumors lacking T cell infiltrates could be sensitized to anti-tumor immunity when suitable ICD inducers, such as oxaliplatin and cyclophosphamide, were combined with anti-PD-1 and/or anti-CTLA-4. They also found that the immune-mediated tumor rejection was due to the direct action of the chemotherapeutic drugs on the tumor cells, was dependent on innate immune sensing through TLR-4 signaling, and required CD8^+^ T cell activity [88]. Second, numerous chemotherapies have been reported to upregulate the expression of PD-L1 on tumor or myeloid cells [86,92,93,94,95], which reduces treatment efficacy but paradoxically increases the target for ICIs that can impede this immunosuppressive effect. Grabosch, et al. demonstrated that cisplatin induced PD-L1 expression on murine ovarian cancer partly via the cGAS/STING pathway and the combination of cisplatin with anti-PD-L1 improved the overall survival of tumor-bearing mice [95].

The ability of some chemotherapeutic agents to increase TAA expression and promote antigen processing and presentation makes them a feasible combination partner for cancer vaccines [4,96]. In one study, recombinant poxvirus vaccine in combination with docetaxel was shown to inhibit tumor growth in a murine colorectal cancer model. The tumor growth suppression was associated with the expansion of vaccine antigen-specific CD8^+^ T cells as well as antigen cascade that generated other T cells specific to vaccine-unrelated antigens [97]. In another study, cisplatin/vinorelbine in combination with a yeast-based vaccine promoted an antigen-specific CD8^+^ T cell response and increased the tumor surface expression of Fas, which increases sensitivity to CTL-mediated killing, thereby improving the survival of non-small-cell lung-cancer-bearing mice [98]. In these studies, chemotherapy also had a direct effect on the immune cells, including regulatory T cell (Treg) depletion and effector T cell expansion, which additionally contributed to the enhanced anti-tumor response [97,98]. 

### 2.3. Chemotherapy-Induced Immunogenic Cell-Stress Response in the Clinic

Chemotherapy in combination with immunotherapy, such as ICIs and other tumor-targeting monoclonal antibodies, has been approved in multiple cancer settings [99,100,101,102]. However, the contribution of chemotherapy-mediated ICD and immunogenic modulation to the immunological response in these combinations is largely unknown. Elucidating the link between immunogenic cell stress response and therapeutic response in the clinic is limited by several factors, including the gap in knowledge with regard to the most effective chemotherapy dose and schedule to induce immunogenic cell stress, lack of systemic biomarkers, and difficulty in obtaining appropriate patient samples for monitoring [103]. In most cases, chemotherapeutic agents are used at or near their maximum tolerated dose, which is effective at inducing tumor cell death but may also result in toxicities [104]. Alternative chemotherapy dosing schedules such as metronomic chemotherapy (i.e., frequent low doses of chemotherapy) and medium-dose intermittent chemotherapy are currently being explored [105,106,107]. Clinical trials identifying ICD-related biomarkers are underway but thus far have given mixed results. For example, in breast cancer, one study showed that chemotherapy was associated with decreased serum HMGB1, which correlated with treatment efficacy [108], while a separate study demonstrated that complete loss of HMGB1 was linked to poor response [109]. In an ongoing study investigating colorectal liver metastases for markers of ICD (NCT01516710), it was observed that patients who had received neoadjuvant chemotherapy (including oxaliplatin) exhibited a gene signature related to toll-like receptor signaling, IFN response, and leukocyte infiltration [110]. However, a follow-up study showed that there was no association between neoadjuvant chemotherapy and intratumoral T cell density within colorectal liver metastases. Investigating further, it appeared that this discrepancy may have been temporal in nature, being associated with a transient increase in T cell density, as there was a significant difference in T cell infiltrate between patients who received neoadjuvant chemotherapy fewer than 9.5 weeks before resection of liver metastases and those who did not receive chemotherapy or those who had a longer interval between treatment and resection [111]. The implications of these findings not only described possible biomarkers but also gave better comprehension that can inform optimal treatment schedules combining chemotherapy and immunotherapy.

Two recent Trial Watch publications identified over 150 ongoing or recently completed clinical trials that included at least one ICD-inducing chemotherapeutic agent [112,113], and we have identified 10 additional trials on the ClinicalTrials.gov database (http://www.clinicaltrials.gov/, accessed on 1 November 2022) examining or utilizing chemotherapy-mediated immunogenic cell-stress responses that were initiated since 2019 (Table 1). In line with the current interest in combining standard-of-care chemotherapy with ICIs, most of these studies employ cyclophosphamide, oxaliplatin, or doxorubicin in combination with anti-PD-1 [78,112,113]. Furthermore, a number of these studies are being applied in the breast cancer setting. The TONIC trial (NCT02499367) aims to identify strategies, including no treatment induction versus radiotherapy, cisplatin, cyclophosphamide, or doxorubicin, that could sensitize metastatic triple negative breast carcinoma (TNBC) to PD-1 blockade [114]. Preliminary reports suggest that the overall objective response rate was 20% and that the majority of responses were observed with doxorubicin, then with cisplatin. Furthermore, RNA analysis revealed an upregulation of gene signatures involved in PD-1/PD-L1 and T cell cytotoxicity pathways after induction with doxorubicin and cisplatin [114]. Furthermore, there was a trend towards improved T cell infiltration and increased T-cell receptor (TCR) diversity in the doxorubicin cohort, which is currently expanded in the TONIC-2 trial (NCT04159818). In two parallel randomized Phase IIb studies, one in metastatic TNBC (ALICE; NCT03164993 [115]) and the other in hormone positive breast cancer (ICON; NCT03409198 [116]), pegylated liposomal doxorubicin and cyclophosphamide are being investigated in combination with atezolizumab and nivolumab plus ipilimumab, respectively. The hypothesis is that the semi-metronomic chemotherapy regimen will induce ICD and counter immunosuppressive cells, thereby sensitizing patients to ICIs. The primary objective of these studies is to investigate the safety and efficacy of adding ICIs to the immunogenic chemotherapeutic regimen. In addition, these studies will include a comprehensive assessment of quality of life, examination of changes in the immunological milieu, analysis of biomarkers, and mechanisms of resistance [115,116]. The results of these trials will inform the development of combinatorial therapeutic interventions involving immunogenic chemotherapies and ICIs.

In the clinic, cancer vaccines have proven safe, although thus far have shown only modest therapeutic efficacy [117]. Several clinical trials have been designed to test the synergistic or additive effect of combining immune-modulating chemotherapies with vaccines. One previously concluded Phase II study assessed docetaxel alone versus docetaxel combined with PANVAC in metastatic breast cancer [118]. PANVAC consists of viral vectors, including a priming dose with vaccinia vector and subsequent boosting doses with fowlfox vector, recombinant for human CEA and MUC-1 genes together with transgenes for a triad of costimulatory molecules B7.1, ICAM-1, and LFA-3 (TRICOM). The docetaxel + PANVAC combination resulted in progression-free survival (PFS) of 7.9 months compared to 3.9 months in the docetaxel arm alone. In the combination arm, 11/16 (69%) evaluable patients developed T cell specific responses to CEA or MUC-1, while in the docetaxel arm alone, 8/15 (53%) patients developed T cell responses to the vaccine-targeted TAAs [118]. This indicated that there was no correlation between generation of T cell specific immune response and time to progression, but rather supported the observation that docetaxel-induced immunogenic modulation was sufficient to mediate the improved outcome in PFS. 

An ongoing clinical trial employing the TRICOM vaccine platform targeting the TAA PSA (PROSTVAC) aimed to evaluate the optimal sequence of PROSTVAC and docetaxel in metastatic castration-sensitive prostate cancer (NCT02649855). The study design is composed of three arms with androgen deprivation therapy (ADT) followed by docetaxel plus PROSTVAC versus ADT, followed by sequential docetaxel then PROSTVAC versus ADT followed by PROSTVAC then docetaxel. Preliminary analysis showed that of the patients who received ADT followed by docetaxel and then PROSTVAC, 31%, 50%, and 50% had CD4/CD8 responses to tumor antigens PSA, MUC-1, and brachyury, respectively. Meanwhile, the cohort that received concurrent docetaxel and PROSTVAC had 50%, 58%, and 42% responses, and the cohort that received PROSTVAC and then docetaxel had 72%, 39%, and 71% responses to PSA, MUC-1, and brachyury, respectively. These preliminary data suggest that scheduling the vaccine followed by chemotherapy generated the most robust immune activation [119]. Associations between immune responses and clinical outcomes are yet to be reported. 

## 3. Radiation and Immunogenic Cell Stress

### 3.1. Mechanisms of Radiation-Induced Immunogenic Cell Stress

While radiation is primarily purposed as a cytotoxic therapy, an immunogenic potential for this therapeutic intervention has also been recognized for more than two decades [120,121]. In 2002, Friedman summarized what was known of this immunogenic potential of radiotherapy, employing Matzinger’s danger model of immunity [122], and made a call-to-action to elucidate the mechanisms of radiation-induced immunogenicity, envisioning that these could be exploited in the field of immuno-oncology [120]. Since then, it has become well-established that radiotherapy-mediated immunogenic cell stress involves the induction of DNA damage and the generation of reactive oxygen species (ROS), effectuating an ER stress response and culminating in the release of DAMPs and/or immunogenic alterations in tumor phenotype [76,123,124,125,126].

The abscopal effect—a rare clinical phenomenon whereby irradiation of a primary lesion leads to regression of a distal lesion outside of the radiation field in metastatic disease—served as early evidence of the immunogenic potential of radiotherapy. In a landmark case report, a patient with metastatic melanoma receiving maintenance ipilimumab experienced surprising and remarkable regression of their metastatic disease following radiotherapy of a paraspinal lesion [127]. Preclinical studies in murine tumor models have been instrumental in providing mechanistic understanding of the contribution of immunogenic cell stress in mediating radiation-induced systemic anti-tumor immunity responsible for the abscopal effect [128]. Utilizing a murine model of mammary carcinoma, Demaria, et al. emphasized that the abscopal effect observed when radiotherapy was combined with a dendritic cell growth factor (Flt3L) was immune-mediated, as it was not recapitulated in nude mice, and antigen-specific, as abscopal regression was not evident in a distal lymphoma lesion of disparate antigenicity [129]. In a study that employed a three-dimensional volume-based lattice radiation modality to deliver high-dose radiation to whole versus partial tumor volumes, it was observed that 20% volume irradiation in two 10% volume lattices mediated equivalent tumor regression as whole volume irradiation in both the primary tumor (i.e., bystander effect) and distal tumor (i.e., abscopal effect) [130]. Furthermore, the tumor regression observed with partial volume irradiation was associated with a robust Th1 immune response, as well as an anti-angiogenic phenotype [130]. Similarly, localized radiotherapy in combination with TAA-targeted vaccine resulted not only in the induction of vaccine-derived TAA-specific T cells but also the activation of T cells against antigens not encoded by the vaccine. This antigen cascade stimulated by radiotherapy and vaccine induced the regression of the primary TAA-expressing tumor, but more remarkably, it also mediated the regression of a distal tumor that was of the same origin as the primary tumor but negative for the TAA targeted by the vaccine [131].

The immunogenic cell stress generated by radiotherapy alters the tumor phenotypic profile, sensitizing the surviving fraction of tumor cells to immune targeting. Radiation, even at sublethal doses, was found to promote surface translocation of CRT, whereas the reduction of surface CRT, either by blockade or siRNA knockdown, abrogated this enhanced effect following radiation exposure [132]. Furthermore, siRNA knockdown of PERK interrupted CRT translocation to the plasma membrane, emphasizing the role of ER stress and CRT translocation in enhancing CTL-mediated lysis of radiation-exposed tumor cells. Radiotherapy is also able to upregulate TAA expression (e.g., CEA and MUC1) and the expression of components of the antigen processing and presentation machinery (MHC-I, peptide transporters, chaperones, and immunoproteasome subunits) [18,132,133,134]. Notably, radiation exposure could diversify the peptide pool being presented by MHC-I on the tumor cell surface, which has profound implications in terms of antigen cascade [134]. Radiotherapy may also shift the balance between pro-apoptotic and anti-apoptotic machinery toward a more precarious state poised for immune-mediated cell death. For example, radiotherapy has been reported to augment Fas and ICAM-1 expression [18,50,133,135]. These molecules may contribute to, but individually are not sole determinants of, radiotherapy-induced immunogenicity, since some radiation-exposed tumor cells that did not upregulate Fas or possessed defective Fas signaling were still susceptible to CTL-mediated killings [18], whereas blocking ICAM-1 did not abrogate CTL-mediated lysis [50]. Nevertheless, in studies employing a murine colorectal cancer model, the sustained upregulation of Fas by radiotherapy in combination with TAA-specific CTL adoptive transfer or vaccine was associated with tumor rejection [50,135]. Furthermore, the anti-tumor efficacy of the radiotherapy and vaccine combination was abrogated in mice defective for Fas signaling, establishing a role for Fas receptor as a mediator of immunogenic cell-stress response following radiotherapy [135]. Lastly, radiotherapy could upregulate costimulatory molecules to facilitate T cell activation. OX40L and 41BBL were reported to increase in three human prostate carcinoma models after radiation exposure, which translated to significantly increased CTL reactivity to target antigen [53].

Overall, these studies confirm that radiotherapy induces cell stress that results in immunogenic modulation and ICD. Similar to what was observed with chemotherapy, these two responses exist on a continuum. A study by Gameiro, et al. observed increasing secretion of ATP and HMGB1 in a dose-dependent manner from sublethal to lethal irradiations. Notably, ATP and HMGB1 were secreted in some tumor models even at sublethal doses of radiation, while CRT surface translocation, classically a cardinal sign of immunogenic cell death, occurred in all tumor models at sublethal doses of radiation [132]. This was corroborated in another study demonstrating significantly enhanced ATP and HMGB1 secretion following sublethal radiation exposure, in conjunction with upregulated immunogenic DAMPs and cytokines [136]. 

### 3.2. Different Modalities of Radiotherapy Induce Immunogenic Cell Stress

**Figure 2:**
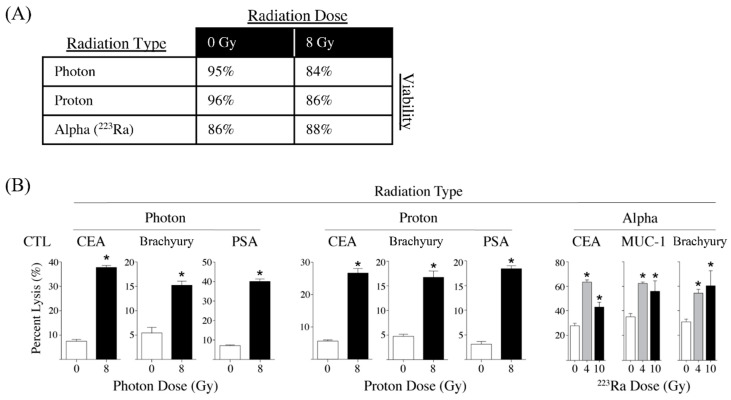
Sublethal exposure to photon, proton, or alpha radiation increases CTL lysis of prostate carcinoma cells in vitro. (**A**) Sublethal exposure of LNCaP prostate tumor cells in vitro to photon, proton radiation, or alpha radiation (223Ra) has minimal effect on cell viability. Human prostate carcinoma cells were mock-irradiated (0 Gy) or treated with either 8 Gy of photon radiation, 8 Gy proton radiation, or 8 Gy of 223Ra. Radiation-treated cells were cultured for an additional 72–96 h, and viability was determined by AO/PI viability dye. Depicted is % viable cells. (**B**) Human LNCaP prostate carcinoma cells were mock-irradiated (0 Gy, open bars) or treated with either 8 Gy of photon radiation (closed bars), 8 Gy proton radiation (closed bars), 4 Gy of of 223Ra (gray bars), or 10 Gy of 223Ra (closed bars). Radiation treated cells were cultured for an additional 72–96 h and then used as targets in an overnight CTL lysis assay. CEA-, MUC-1-, and brachyury-specific CD8+ T cells were used as effectors at an E:T ratio of 30:1. Experiments were repeated 1–3 times with similar results. * = *p* < 0.05. Adapted from [132,140,141].

As the field of radiation oncology evolves with more diverse and advanced modes of radiation delivery [137], the question arises whether each of these different radiation modalities is capable of inducing immunogenic cell stress in the same way as has been described in the above studies, each of which employed photon radiation sources (Figure 2). Radiotherapy involving beta (β^−^) particle emitters has been shown to induce immunogenic cell stress responses. After exposure to β^−^ particle emission using Sm-153, a bone-seeking radionuclide used to palliate metastatic bone pain, different human tumor cell lines demonstrated upregulation of at least two of the following markers: Fas, MHC-1, ICAM-1, CEA, and MUC-1 [133]. Sm-153 exposure also resulted in enhanced CTL-mediated lysis specific for MUC-1, CEA, and PSA. In another study employing a β^−^ particle emitter, anti-CEA monoclonal antibody radiolabeled with yttrium-90 (Y-90) was combined with CEA-targeted vaccine to treat mice implanted with CEA+ murine carcinoma tumors [138]. This combination resulted in increased survival of tumor-bearing mice when compared to either modality alone. This survival advantage was mediated by the engagement of the Fas/Fas ligand pathway and was also associated with antigen cascade and increased tumor infiltration of CEA-specific T cells. Interestingly, this study further showed that the tumor-infiltrating CD8^+^ T cells were less radiation-sensitive than naïve CD8^+^ T cells. This observation was corroborated by an earlier study, which demonstrated that memory CD8^+^ T cells were more resistant to apoptosis than naïve CD8^+^ T cells following whole-body irradiation in a murine model [139]. 

Compared to β^−^ particle radiation, alpha particle radiation has a shorter path length and larger linear energy transfer, which translates to less myelosuppression in the adjacent bone marrow compartment [142]. When Ra-223, an alpha particle emitter that complexes to metastatic bone lesions, was used to irradiate different human carcinoma cell lines, it was observed that Ra-223 induced ER stress and upregulated MHC-I and CRT expression [138]. Moreover, exposure to Ra-223 enhanced CTL-mediated lysis, which was dependent upon CRT surface expression, as this effect was abrogated with the addition of calreticulin-blocking peptide. 

Proton particle radiation is a new and impressive radiation modality that is capable of delivering high doses of radiation in a precise and accurate manner such that adjacent healthy tissues can be spared, as in cases of spinal cord or skull base tumors [139]. In a study using a diverse set of human cancer cell lines, it was demonstrated that proton particle radiation could upregulate MHC-1, ICAM-1, and TAAs, as well as surface translocation of CRT [137]. Proton particle radiation also mediated enhanced CTL-mediated lysis, which was dependent on CRT. Intriguingly, the study further demonstrated that resident cancer stem cells were more viable following radiotherapy than non-cancer stem cells yet maintained upregulation of calreticulin, thus suggesting that while cancer stem cells are more recalcitrant to the cytotoxic effects of radiotherapy, they remain susceptible to radiation-induced immunogenic cell stress. 

Radiofrequency ablation (RFA) is a type of thermal ablation modality [143], as opposed to ionizing radiation (IR), as illustrated by the examples above. When sublethal hyperthermia was applied to a CEA-expressing murine colorectal carcinoma in vitro, it induced Fas, MHC-I, and CEA upregulation, which translated to enhanced CTL-mediated lysis [144]. In vivo, RFA also induced immunogenic modulation, and when combined with CEA-targeting vaccine, promoted tumor regression and abscopal effect, which was associated with CD4 immune responses to CEA and cascade antigens [144].

Another non-ionizing radiotherapy that induces immunogenic cell stress is phototherapy [7,8]. Phototherapy destroys cancer cells by utilizing light to trigger photosensitizers that produce ROS (photodynamic therapy) or photothermal agents that generate heat (photothermal therapy) [145,146]. When appropriate photoagents and light doses are employed, phototherapies can promote the release of TAAs and DAMPs [8,147]. This immunogenic cell stress response can be further exploited to enhance antitumor activity through combination with immunotherapy, including checkpoint blockade, metabolic modulators, targeted antibodies, and CAR T cells (reviewed here: [148,149]). Furthermore, immunotherapy and phototherapy can be integrated such that monoclonal antibodies that recognize tumor antigens are conjugated with photoagents (ex. IR700), allowing for highly targeted photoimmunotherapy (PIT) that is activated by exposure to near infrared light (NIR) [150]. A preclinical study utilizing avelumab, an anti-PD-L1 antibody, that is conjugated with IR700 (avelumab-IR700) showed specific binding and killing of tumor cells after exposure to near-infrared light [151]. Importantly, NIR-PIT using avelumab-IR700 was shown to suppress tumor growth and improve survival in a lung adenocarcinoma model. In the clinic, several trials investigating EGFR-targeting cetuximab-IR700 (RM1929) in head and neck cancer are underway (NCT05265013, NCT05182866, NCT03769506, NCT04305795). 

Finally, stereotactic ablative radiotherapy (SABR) represents a technological advancement in the field of radiation oncology that can deliver high doses of photon radiotherapy with high precision and accuracy [152]. Along with these advances come questions regarding optimal scheduling, dosing, and fractionation in order to maximally induce and, in turn, exploit the resulting immunogenic cell stress (reviewed here: [153]). 

### 3.3. Radiotherapy-Induced Immunogenic Cell Stress Response in the Clinic

Observations from several early studies have lent credence to the immunogenic potential of radiotherapy in the clinic. Select ongoing studies on the ClinicalTrials.gov database (http://www.clinicaltrials.gov/, Accessed on 1 November 2022) examining the immunological aspect of radiotherapy are listed in Table 2. In a study by Nesslinger, et al. comparing pre- and post-treatment serum samples in patients with nonmetastatic prostate cancer, it was demonstrated that 4 of 29 (13.8%) patients receiving external beam radiotherapy and 5 of 20 (25%) patients receiving brachytherapy developed antibody responses to TAAs following treatment, as compared to 0 of 14 (0%) patients receiving radical prostatectomy and 2 of 36 (5.6%) patients who chose watchful waiting, serving as control [154]. Meanwhile, Schaue, et al. demonstrated that survivin-specific CD8^+^ T cells were increased in 9 of 13 (69%) colorectal cancer patients and in 7 of 11 (64%) prostate cancer patients receiving chemoradiotherapy and radiotherapy, respectively [155]. Collectively, these early observations suggested that standard-of-care radiotherapy had the capacity to elicit a TAA-specific immune response. An ongoing observational clinical study in nonmetastatic prostate cancer aims to characterize the immunomodulatory effects of radiotherapy on the T cell, NK cell, B cell, Treg, and Breg compartments (NCT04774133), which will further elucidate the immunogenic potential of radiotherapy. 

Clinical trials examining the capability of enhancing TAA-specific immune responses using vaccines in combination with radiotherapy have previously been undertaken. Radiotherapy was combined with PROSTVAC in a randomized Phase II clinical study in patients with localized prostate cancer, and investigation of TAA-specific immune responses demonstrated at least a three-fold increase in PSA-specific T cells in 13 of 17 patients receiving radiotherapy and vaccine, as compared to no detectable increases in 11 patients receiving radiotherapy alone [156]. Furthermore, antigen cascade was observed in six of eight evaluable patients, with TAA-specific T cell responses to PSMA, PAP, PSCA, and/or MUC-1. Another Phase II trial utilizing PROSTVAC, this time in combination with Sm-153, in patients with metastatic prostate cancer with bone lesions having failed prior docetaxel therapy demonstrated a significant increase in progression-free survival to 3.7 months with radiation and vaccine, as compared to 1.7 months with radiation alone (*p* = 0.041; HR = 0.51, *p* = 0.046) [157]. Collectively, these studies challenged the traditional dogma that radiotherapy was immunosuppressive and demonstrated that radiotherapy can be combined with vaccines.

A study employing intratumoral injection of poly-ICLC (TLR3 agonist) in combination with an autologous dendritic cell vaccine in heavily pre-treated patients with progressive metastatic solid tumors saw 5 of 10 patients achieve stable disease as their best response [158]. Remarkably, when a second cohort was recruited with the added combination of SABR, five of six patients achieved stable disease as their best response, illustrating a rational partnership between DC vaccine and radiotherapy. There are ongoing clinical trials exploring this rational partnership employing external beam radiotherapy in combination with autologous dendritic cell adoptive cell transfer and pneumococcal vaccine (to induce a Th1 milieu) in patients with unresectable liver cancer (NCT03942328) and another employing subtherapeutic radiotherapy in combination with intratumoral injection of poly-ICLC (TLR3 agonist) and Flt3L (dendritic cell growth factor), in addition to systemic pembrolizumab (anti-PD-1) in patients with non-Hodgkin’s lymphoma, head and neck squamous cell carcinoma, or metastatic breast cancer (NCT03789097).

In a study investigating the combination of radiotherapy and ipilimumab in metastatic melanoma, as best response, 5 of 22 (22.7%) patients achieved partial response and 3 of 22 (13.6%) achieved stable disease [159]. Remarkably, of the patients who progressed and received salvage pembrolizumab, 6 of 12 (50%) responded favorably with long-term survival. In an interim analysis of this clinical trial, Victor, et al. employed the B16-F10 murine melanoma model and demonstrated in the group receiving combination radiotherapy and anti-CTLA4 that distal lesions resistant to abscopal regression had marked upregulation of PD-L1 as a dominant mechanism mediating resistance [160]. Accordingly, they demonstrated that resistance to abscopal regression was surmounted with the addition of PD-1/PD-L1 axis blockade, and that the triple combination was superior to dual checkpoint blockade without radiotherapy. Analysis of tumor-infiltrating lymphocytes (TILs) implicated that anti-CTLA4 predominantly mediated a decrease in the Treg compartment, whereas anti-PD-L1 promoted a strong increase in CD8^+^ TILs. Moreover, the combination synergistically increased the CD8^+^/Treg ratio. Finally, while radiation had a small positive impact on CD8^+^ TILs, the predominant effect brought on by radiation was in diversifying TCR clonotypes, again demonstrating the impact of antigen cascade as a consequence of immunogenic cell stress.

Based on these observations, an ongoing Phase II clinical trial was launched in the setting of metastatic melanoma to investigate the triple combination of radiotherapy plus ipilimumab plus nivolumab (NCT03646617). There is also another ongoing study in the setting of metastatic non-small-cell lung cancer and head-and-neck squamous-cell carcinoma investigating the combination of immune checkpoint inhibition (pembrolizumab or nivolumab or atezolizumab) followed by radiotherapy within 14 days, hypothesizing that a robust effector immune response active at the time of immunogenic cell stress induction with radiotherapy will best exploit this combination (NCT03313804). Another group is investigating the safety and efficacy of different radiotherapy schedules in combination with pembrolizumab in an ongoing clinical trial in the setting of early/operable breast cancer (NCT04454528). All patients will undergo surgery on day 0: arm one will receive radiotherapy on day -14 followed by pembrolizumab on day -7; arm two will receive the reverse schedule; arm three will receive only pembrolizumab on day -14; and arm four will not receive neoadjuvant therapy.

## 4. Small-Molecule Inhibitors and Immunogenic Cell Stress

### Mechanisms of SMI-Induced Immunogenic Cell Stress

Like chemotherapy and radiation, select SMIs have been reported to promote immunogenic cell stress responses [2]. SMIs are drugs, usually less than 500 Da in size, that can target both extracellular and intracellular proteins including receptors, kinases, epigenetic regulatory proteins, DNA damage repair enzymes, and proteasomes [161,162]. As of December 2020, 89 SMIs have been approved in the US and in China for application in different cancer settings [162]. 

One of the SMIs best described to induce immunogenic cell stress is bortezomib, a proteasome inhibitor approved for the treatment of multiple myeloma (MM) and mantle cell lymphoma (MCL) [163]. As a proteosome inhibitor, bortezomib causes the accumulation of unfolded proteins and increases ER stress [164]. Accordingly, bortezomib has been shown to promote the surface translocation of CRT and HSP90, increasing DC phagocytosis of dying MM cells, which in turn was shown to increase effector memory CD4+ and CD8+ T cell responses in co-culture [165,166]. CRT surface translocation was required for the induction of immune response, as the knockout of the CRT gene in MM cells abolishes the efficacy of the bortezomib-killed cells to protect against tumor rechallenge in immunocompetent mice. Furthermore, bortezomib induced the expression of ICD-related genes, most of which were identified as IFN-stimulated genes, in the CRT-expressing cells but not in CRT-deficient ones. Moreover, the type-1 IFN response induced by bortezomib was demonstrated to be mediated by the cGAS/STING pathway [166]. This study implicates a broader potential for bortezomib in mediating anti-tumor effects, especially in combination with immunotherapy. Currently, this proteasome inhibitor is being evaluated with ICIs (Table 3).

Poly (ADP-ribose) polymerase (PARP) inhibitors prevent the repair of single-strand DNA breaks and have been shown to cause synthetic lethality in homologous recombination repair deficient tumors, such as those with BRCA mutations [167]. As such, PARP inhibitors have been approved for the treatment of BRCA-mutated ovarian, breast, and pancreatic cancer. In addition to inducing synthetic lethality, PARP inhibitors have recently been shown to induce immunogenic modulation. In vitro studies showed that the PARP inhibitor olaparib upregulated surface expression of Fas and TRAIL-R2 [168,169]. In turn, olaparib-treated prostate cancer cells were more susceptible to NK cell-mediated killing, and knocking out TRAIL-R2 was shown to abrogate this enhanced effect [169]. In a preclinical study, the PARP inhibitor veliparib in combination with ionizing radiation significantly increased MHC-I and PD-L1 expression on murine colorectal cancer cells compared to radiation or veliparib alone [170]. However, the addition of veliparib did not improve radiation-induced calreticulin surface translocation, indicating that radiation was the main inducer of ER stress in this combination. Nevertheless, the combination of veliparib, radiation, and PD-1 blockade delayed tumor growth and prolonged survival in tumor-bearing mice compared to any other permutation this triple therapy tested. However, the contribution of veliparib-induced immunogenic modulation to the therapeutic benefit remains to be elucidated. Clinical trials assessing PARP inhibitors with ICIs have recently been reviewed by Wu et al. [171].

Different SMIs that are being applied as endocrine therapy in patients with hormone-sensitive breast and prostate cancer [172] have been shown to promote immunogenic modulation. Androgen-receptor (AR) antagonists, enzalutamide, and abiraterone sensitized murine and human prostate tumor cells to T cell-mediated lysis [173,174]. The immunomodulatory capacity of these antagonists was associated with the downregulated anti-apoptotic molecule neuronal apoptosis inhibitory protein (NAIP) in AR+ cells [173] and upregulated cell surface expression of Fas and MHC-1 [174]. Combinatorial treatment in the spontaneous prostate cancer mouse model TRAMP with enzalutamide and TAA-targeting yeast-based vaccine improved overall survival compared to control mice or mice receiving vaccine or enzalutamide alone [174]. Furthermore, the combination was shown to induce antigen cascade, broadening the immunological response. Although AR antagonists are normally used as a standard of care for prostate cancer, androgen deprivation agents have additionally shown anti-tumor efficacy against breast cancer in preclinical studies as well. Enzalutamide and abiraterone were shown to sensitize breast cancer cells to CTL-mediated lysis independent of detectable AR expression [175]. This effect was linked to increased cell surface expression of TRAIL-R1/R2, as well as decreased expression of osteoprotegerin (OPG). Traditionally, OPG functions to inhibit osteoclastogenesis during bone remodeling, but it was shown in this model that OPG served as a soluble decoy receptor for TRAIL and, accordingly, inhibited TRAIL-mediated apoptosis. Thus, downregulation of OPG in the context of antigen deprivation mitigated this antagonistic effect and enhanced TRAIL-mediated apoptosis [175]. 

Fulvestrant (selective estrogen degrader (SERD)) and tamoxifen (selective estrogen receptor modulator (SERM)) are two additional endocrine deprivation therapies that mediate estrogen receptor antagonism and have been shown to promote immunogenic modulation in triple-negative breast cancer [176]. Treatment with fulvestrant and the tamoxifen metabolite 4-OHT upregulated Fas and TRAIL-R1/R2 and resulted in increased NK cell-mediated lysis of breast cancer cells regardless of estrogen receptor (ER) status [176]. Furthermore, RNA analysis identified G-protein-coupled receptor for estrogen (GPR30) as a putative player in the immunogenic modulation induced by fulvestrant and 4-OHT. Targeted activation of GPR30 using its specific agonist, G-1, resulted in increased NK cell killing, while the knockdown of this receptor abrogated the NK cell killing mediated by fulvestrant and 4-OHT [176]. Finally, the combination of fulvestrant with the IL-15 superagonist, N803, which has been shown to enhance NK cell activity [177], was found to result in superior anti-tumor activity in a TNBC murine model. Overall, these preclinical studies demonstrate the immunogenic potential of endocrine deprivation therapy using SMIs. Some of the clinical trials on the ClinicalTrials.gov database (http://www.clinicaltrials.gov/, Accessed on 1 November 2022) that opened since January 2020 combining endocrine deprivation therapy with immunotherapy are listed in Table 3.

## 5. Conclusions

The ability of radiotherapies, chemotherapies, and SMIs to induce ICD and immunogenic modulation serves as an important anti-cancer modality that may be able to enhance or complement the activity of other anti-cancer treatments, specifically immunotherapy. In fact, combination therapy with chemotherapy and immune checkpoint blockade is already approved in several indications [99]. Moreover, some clinical studies combining conventional therapy and immune checkpoint blockade have demonstrated improved overall survival [178,179]. The role of immunogenic cell stress in the clinical response in these combination therapies remains largely unknown and it can be expected that the application of combination therapy with conventional therapeutic agents and immune checkpoint blockade may be expanded and improved as more mechanistic data emerge.

Ongoing clinical studies combining chemotherapy, radiotherapy, and SMIs with immunotherapy involve examining the changes in immune cell milieu and will give important data on the synergistic/additive immunological effects of conventional and/or targeted therapies with immunotherapies. Furthermore, some of these studies will help elucidate the appropriate dosage and sequence of these anti-cancer agents. Promising results have already been obtained; however, a more detailed investigation on the collaboration of immunogenic cell stress with immunotherapy will contribute to improving the formulation of combinatorial therapies that result in superior clinical benefits. 

## Figures and Tables

**Table 1:** Clinical trials investigating immunogenic cell stress mediated by chemotherapy.

Clinical Trial	Trial Title	Conditions	Treatment	Phases
NCT03801304	Trial to Evaluate Safety and Efficacy of Vinorelbine With Metronomic Administration in Combination With Atezolizumab as Second-line Treatment for Patients With Stage IV Non-small Cell Lung Cancer	Non-small-cell lung cancer	Atezolizumab Vinorelbine	Phase II
NCT04159818	Immune Induction Strategies to Improve Response to Immune Checkpoint Blockade in Triple Negative Breast Cancer (TNBC) Patients (TONIC-2)	Metastatic breast cancer	Nivolumab Cisplatin Low-dose doxorubicin	Phase II
NCT04043195	Nivolumab and Ipilimumab in Combination With Immunogenic Chemotherapy for Patients With Advanced NSCLC	Advanced non-small cell lung cancer (NSCLC)	Oxaliplatin Nivolumab Ipilimumab	Phase I Phase II
NCT04463368	Isolated Hepatic Perfusion in Combination With Ipilimumab and Nivolumab in Patients With Uveal Melanoma Metastases (SCANDIUM II)	Uveal melanoma Liver metastases	Melphalan Ipilimumab Nivolumab	Phase I
NCT04072263	Adoptive T Cell Therapy in Patients With Recurrent Ovarian Cancer (OVACURE)	Recurrent ovarian cancer	Tumor-infiltrating lymphocytes Interferon alfa 2A Carboplatin Paclitaxel	Phase I Phase II
NCT04262687	Chemotherapy and Immunotherapy as Treatment for MSS Metastatic Colorectal Cancer with High Immune Infiltrate (POCHI)	Metastatic colorectal cancer High immune infiltrate Microsatellite stable (MSS)	Capecitabine Oxaliplatin Bevacizumab Pembrolizumab	Phase II
NCT05420584	Neoadjuvant Arterial Embolization Chemotherapy Combined PD-1 Inhibitor for Locally Advanced Rectal Cancer (NECI)	Rectal neoplasms	Tislelizumab Capecitabine Oxaliplatin	Phase II
NCT04989218	Durvalumab and Tremelimumab with Platinum-based Chemo- therapy in Intrahepatic Cholangiocarcinoma (ICC)	Cholangiocarcinoma	Gemcitabine Cisplatin Tremelimumab Durvalumab	Phase I Phase II
NCT05144698	RAPA-201 Therapy of Solid Tumors	Breast cancer Small cell and non-small cell lung cancer Triple negative breast cancer Gastric cancer Esophageal adenocarcinoma Gastric junction adeno- carcinoma Esophageal squamous cell carcinoma Head and neck cancer Squamous cell carcinoma of oral cavity Squamous cell carcinoma of larynx Squamous cell carcinoma of nasopharynx Squamous cell carcinoma of other specified sites of skin Carcinoma of unknown primary Bladder cancer Malignant melanoma	RAPA-201 cells Carboplatin Paclitaxel	Phase II
NCT05307198	Rectal Artery Infusion Chemotherapy Combined with Anti-PD1 Antibody for MSS LARC (RAIC)	Rectal neoplasms	Capecitabine Oxaliplatin Sintilimab	Phase II
NCT02499367	Nivolumab After Induction Treatment in Triple-negative Breast Cancer (TNBC) Patients (TONIC)	Breast cancer	Nivolumab Radiation therapy Low dose doxorubicin Cyclophosphamide Cisplatin	Phase II
NCT03409198	Phase IIb Study Evaluating Immunogenic Chemotherapy Combined with Ipilimumab and Nivolumab in Breast Cancer (ICON)	Breast cancer Hormone receptor positive tumor Metastatic breast cancer	Ipilimumab Nivolumab Pegylated liposomal doxorubicin Cyclophosphamide	Phase II
NCT03164993	Atezolizumab Combined with Immunogenic Chemotherapy in Patients with Metastatic Triple-negative Breast Cancer (ALICE)	Breast cancer Triple-negative breast cancer	Atezolizumab Pegylated liposomal doxorubicin Cyclophosphamide	Phase II
NCT02649855	Docetaxel and PROSTVAC for Metastatic Castration-Sensitive Prostate Cancer	Prostate cancer Prostate neoplasms	PROSTVAC-V PROSTVAC-F Docetaxel	Phase II

PD-1: programmed cell death protein 1, RAPA-201: rapamycin-resistant T cells, PROSTVAC: vaccine targeting prostate-specific antigen.

**Table 2:** Clinical trials investigating immunogenic cell stress mediated by radiotherapy.

Clinical Trial	Trial Title	Conditions	Treatment	Phases
NCT04774133	The Immunodynamic Effect of Radiotherapy in Prostate Cancer Patients	Prostate cancer	Radiation	N/A
NCT03942328	Modified Immune Cells (Autologous Dendritic Cells) and a Vaccine (Prevnar) After High-Dose External Beam Radiation Therapy in Treating Patients With Unresectable Liver Cancer	Hepatocellular carcinoma Intrahepatic cholangiocarcinoma	External beam radiation, therapeutic autologous dendritic cells, Pneumococcal 13-valent conjugate vaccine	Phase I
NCT03789097	Vaccination With Flt3L, Radiation, and Poly-ICLC	Non-Hodgkin’s lymphoma Metastatic breast cancer Head-and-neck squamous-cell carcinoma	Subtherapeutic radiation Fl3tL Poly-ICLC Pembrolizumab	Phase I Phase II
NCT03646617	Ipilimumab and Nivolumab With or Without Hypofractionated Radiotherapy in Patients With Metastatic Melanoma (RadVax)	Metastatic melanoma	Hypofractionated radiation Ipilimumab Nivolumab	Phase II
NCT03313804	Priming Immunotherapy in Advanced Disease With Radiation	Non-small-cell lung cancer Head-and-neck squamous-cell carcinoma	Stereotactic body radiation or fractionated radiation Nivolumab or pembrolizumab or atezolizumab	Phase II
NCT04454528	BreastVAX: Radiation Boost to Enhance Immune Checkpoint Blockade Therapy (BreastVAX)	Breast cancer	Hypofractionated radiation Pembrolizumab	Phase I Phase II

Flt3L: an immune cell growth factor, Poly-ICLC: immune activating factor.

**Table 3:** Clinical trials investigating the combination of small molecule inhibitors with immune checkpoint blockade.

Clinical Trial	Trial Title	Conditions	Treatment	Phases
NCT04265872	Bortezomib Followed by Pembrolizumab and Cisplatin in metTNBC	Breast cancer	Bortezomib Pembrolizumab Cisplatin	Phase 1
NCT04258683	A Study of Pembrolizumab Added to the Standard First-Line Therapy of Cyclophosphamide, Bortezomib, and Dexamethasone (CyBorD) for NDMM NTE	Multiple myeloma	Cyclophosphamide Bortezomib Dexamethasone Pembrolizumab	Phase 2
NCT04191096 NCT04934722	Efficacy and Safety of Pembrolizumab (MK-3475) Plus Enzalutamide Plus Androgen Deprivation Therapy (ADT) Versus Placebo Plus Enzalutamide Plus ADT in Participants With Metastatic Hormone-Sensitive Prostate Cancer (mHSPC) (MK-3475-991/KEYNOTE-991)	Metastatic hormone-sensitive prostate cancer	Enzalutamide Pembrolizumab	Phase 3
NCT04471974	ZEN-3694, Enzalutamide, and Pembrolizumab for the Treatment of Metastatic Castration-Resistant Prostate Cancer	Castration-resistant prostate carcinoma Metastatic prostate adeno- carcinoma Metastatic prostate small cell carcinoma Stage IV/IVA/IVB prostate cancer AJCC v8	ZEN-3694 Enzalutamide Pembrolizumab	Phase 2
NCT04946370	Maximizing Responses to Anti-PD1 Immunotherapy With PSMA-targeted Alpha Therapy in mCRPC	Prostate cancer	225Ac-J591 Pembrolizumab Androgen receptor pathway inhibitor	Phase 1 Phase 2
NCT04262154	Study of Abiraterone Acetate, Atezolizumab, GnRH Analog and Radiation Therapy in Men With Newly Diagnosed Hormone- sensitive Prostate Cancer	Metastatic prostate cancer	Atezolizumab Abiraterone acetate Prednisone Lupron® (leuprolide) SBRT Enzalutamide	Phase 2
NCT04190056	Pembrolizumab and Tamoxifen With or Without Vorinostat for the Treatment of Estrogen Receptor Positive Breast Cancer	Breast cancer	Pembrolizumab Tamoxifen Vorinostat	Phase 2

225Ac-J591: alpha-emitter actinium-225 conjugated to the anti-PSMA antibody J591; NDMM: newly diagnosed multiple myeloma; NTE: not transplant eligible; SBRT: stereotactic body radiotherapy; ZEN-3694: BET bromodomain inhibitor.

## Data Availability

Not applicable.
